# Photoluminescence response of colloidal quantum dots on VO_2_ film across metal to insulator transition

**DOI:** 10.1186/1556-276X-9-612

**Published:** 2014-11-13

**Authors:** Sergey N Kuznetsov, Alexander B Cheremisin, Genrikh B Stefanovich

**Affiliations:** 1 Physico-Technical Department, Petrozavodsk State University, Lenin av. 33, 185910 Petrozavodsk, Russian Federation

**Keywords:** Metal to insulator transition, Vanadium dioxide, Colloidal quantum dots

## Abstract

We have proposed a method to probe metal to insulator transition in VO_2_ measuring photoluminescence response of colloidal quantum dots deposited on the VO_2_ film. In addition to linear luminescence intensity decrease with temperature that is well known for quantum dots, temperature ranges with enhanced photoluminescence changes have been found during phase transition in the oxide. Corresponding temperature derived from luminescence dependence on temperature closely correlates with that from resistance measurement during heating. The supporting reflectance data point out that photoluminescence response mimics a reflectance change in VO_2_ across metal to insulator transition. Time-resolved photoluminescence study did not reveal any significant change of luminescence lifetime of deposited quantum dots under metal to insulator transition. It is a strong argument in favor of the proposed explanation based on the reflectance data.

**PACS:**

71.30. + h; 73.21.La; 78.47.jd

## Background

The so-called correlated oxides attract much attention due to their prospective applications in electronic and optoelectronic devices (see [1] for review). Vanadium dioxide is the most known among them according to its near room temperature metal-insulator transition (MIT) at approximately 67°C. Nowadays, a tendency arises to couple VO_2_ with other materials that will promote a search for new application fields. For example, phase transition in VO_2_ provides a tunability of localized plasmon resonance in metallic sub-wavelength structures [2]. The incorporation of photoluminescence (PL) ions into VO_2_ could result in a novel photonic material capable of simultaneous optical switching and amplification [3]. In the bilayer structure of ZnO/VO_2_, MIT in VO_2_ was shown to control a photoluminescence response of zinc oxide [4]. To our opinion, it is of interest to study another combination of VO_2_ with highly luminescent fluorophores such as colloidal quantum dots (QDs). In contrast to [4], a deposition of self-assembled QD layer on a VO_2_ film does not require a high-temperature treatment; therefore, the intrinsic properties of vanadium dioxide will be unchanged. In addition, due to QD's small diameter less than 10 nm and amorphous layer structure, the deposited coating is tightly bound with coarse polycrystalline VO_2_ film. This circumstance may stimulate more strong interaction between QDs and VO_2_. In this letter, we report on first observation the PL response of core/shell CdSe/ZnS layer deposited on the VO_2_ film when the latter is passing MIT. Based on well-known intrinsic properties of these QDs, some features in PL response have been revealed in the course of phase transition of the underlying VO_2_ film. Using the unified mathematical treatment of electrical and optical temperature dependencies, we derive and compare characteristic parameters for the system studied. From the comparison, the leading role of MIT in the oxide component of hybrid structure is evident. Besides, our finding provides the useful method to probe MIT in the correlated oxides.

## Methods

Vanadium dioxide films were grown by reactive DC magnetron sputtering on polished sitall (Russian brand of glass ceramics) substrates. Deposition was carried out with AJA International sputtering system. First, the substrates were cleaned with RF etching in Ar. Before deposition, pure vanadium target was cleaned in optimal DC regime at 200 W in a mixture of Ar and O_2_ (flow rates of 14 and 2 sccm, respectively, total pressure of 5 mTorr) while the target was closed by a shutter. After the above preliminary procedures, the shutter was turned off and a deposition was carried out for 20 min on the sitall substrate at room temperature. Immediately after deposition, a film was annealed at 520°C for 40 min in pure O_2_ at a pressure of 10 mTorr. These conditions provide the deposition of vanadium dioxide layer with approximately 150 nm in thickness. Fabricated VO_2_ films were polycrystalline with rather wide dispersion in grain sizes. Mean grain dimension was near 90 nm as revealed by AFM measurement in contact mode.

**Figure 1:**
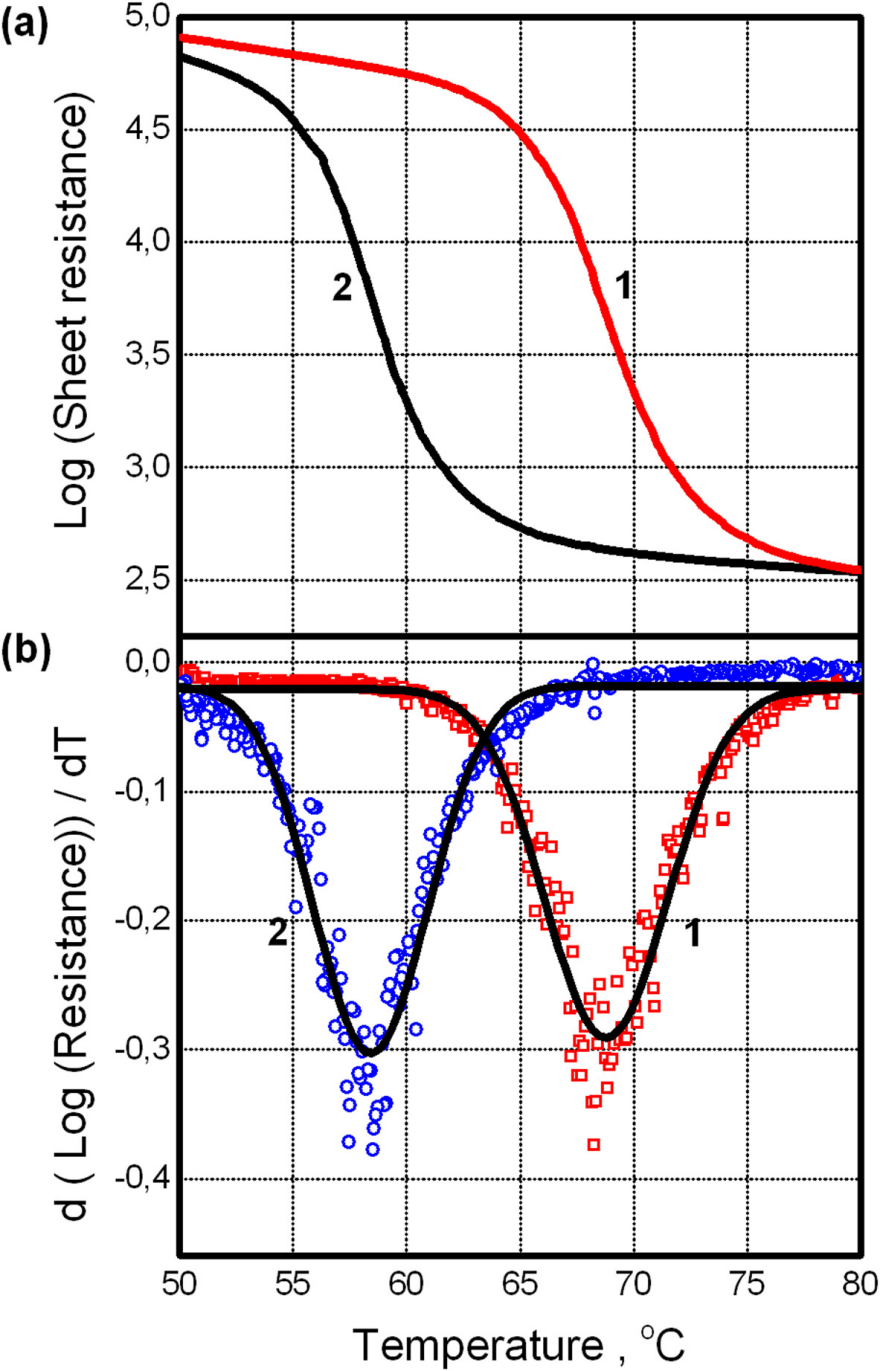
**Electrical response of VO**_**2 **_**film across metal to insulator transition. (a)** Decimal logarithmic plot of sheet resistance of VO_2_ for heating and cooling (curves 1 and 2, respectively). **(b)** Corresponding derivatives (symbols) and their Gaussian fit (lines).

**Figure 2:**
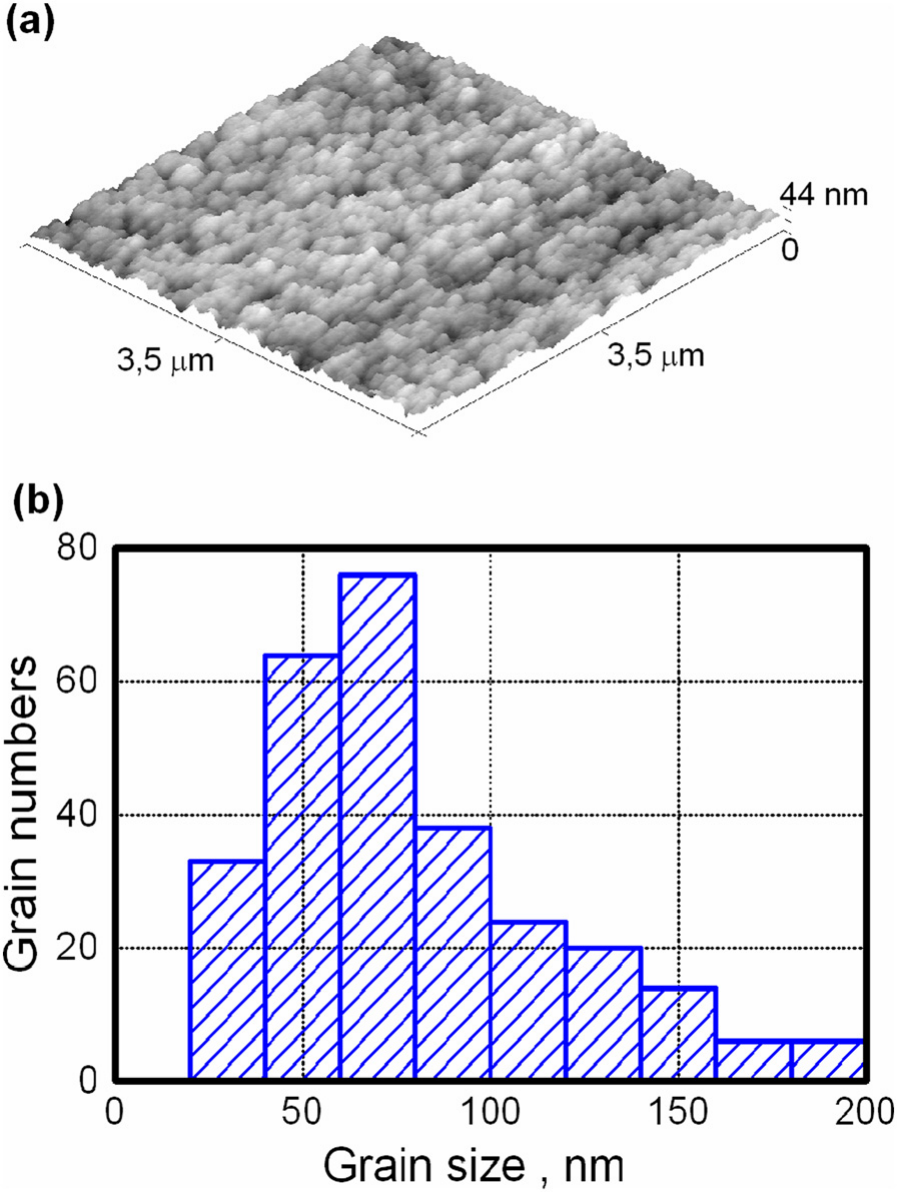
**AFM image (a) and extracted histogram of grain size distribution (b) in polycrystalline VO**_**2 **_**film.**

Electrical properties of VO_2_ under study were obtained with four-point conductance method. In the temperature range from RT to approximately 100°C, a sheet resistance varied more than 2 orders of magnitude (Figure 1a). It should be stressed that our preparation conditions provided the fabrication of VO_2_ on c-plane sapphire with resistance change more than 3 orders. It is indicative for good quality near stoichiometric VO_2_ films. Unfortunately, the present study could not be carried out on sapphire substrates due to some experimental limitations. For heating direction, the temperature of MIT transition has a standard magnitude of 67 ± 1°C. Note that for its estimation, we used a logarithmic linearization of sheet resistance. Then, the first derivative was found with the following fitting of Gaussian curve [5] - see Figure 1b. As shown from Figure 1, the electrical hysteresis loop for our films is rather wide which is approximately 10°C that correlates with broad grain size distribution from AFM data (Figure 2a,b). Such a correlation was earlier observed in [6]. The transition regions for heating and cooling branches were estimated as full width on half maximum (FWHM) of Gaussian curves.

In the second stage of the present study, the VO_2_ films were decorated with colloidal core/shell quantum dots by spin-coating. CdSe/ZnS dots were obtained from Evident Technologies. They consist of CdSe core with diameter of ≈ 5 nm and ZnS shell ≈ 2 nm thick. These QDs are characterized by high quantum efficiency PL of 90% at a peak wavelength of approximately 630 nm and high stability against moderate heating up to approximately 100°C. QDs were spin-coated from a dilute solution in toluene on the VO_2_ film at approximately 2000 rpm. After drying in the air at room temperature, the obtained layers were baked at 100°C in the air for 1 h. This procedure served to minimize an irreversible change of photoluminescence response in further heating/cooling cycles. QD layer thickness was ≈ 50 nm as revealed by a profilometer. PL measurements were carried out under UV excitation in narrow band centered at 385 nm (Nichia UV-LED) with excitation power density of approximately 1 mW/cm^2^. PL spectra were recorded by grating spectrometer with TE-cooled CCD camera and were corrected for spectral response. For temperature-dependent PL measurement, a sample was mounted in homemade temperature cell. A temperature change rate under heating/cooling was not constant, but its value did not exceed 10°C/min. According to [7], there is no significant shift in phase transition parameters of VO_2_ at this rate. It should be noted that a temperature was recorded by a thermopile aligned to the outer surface of the VO_2_ film. We estimate an uncertainty in absolute temperature of a sample which does not exceed ±1°C. PL decay measurements were performed on epifluorescence microscope. The excitation source was a pulsed laser diode operating at 405 nm with 300 ps pulse duration and 4 MHz repetition rate. QD luminescence is collected using the same microscope objective with band-pass filter and detected by avalanche photodiodes operated in Geiger mode. Lastly, transient emission dynamics are analyzed by a time-correlated single-photon counting module. The overall temporal resolution was better than 100 ps.

## Results and discussion

**Figure 3:**
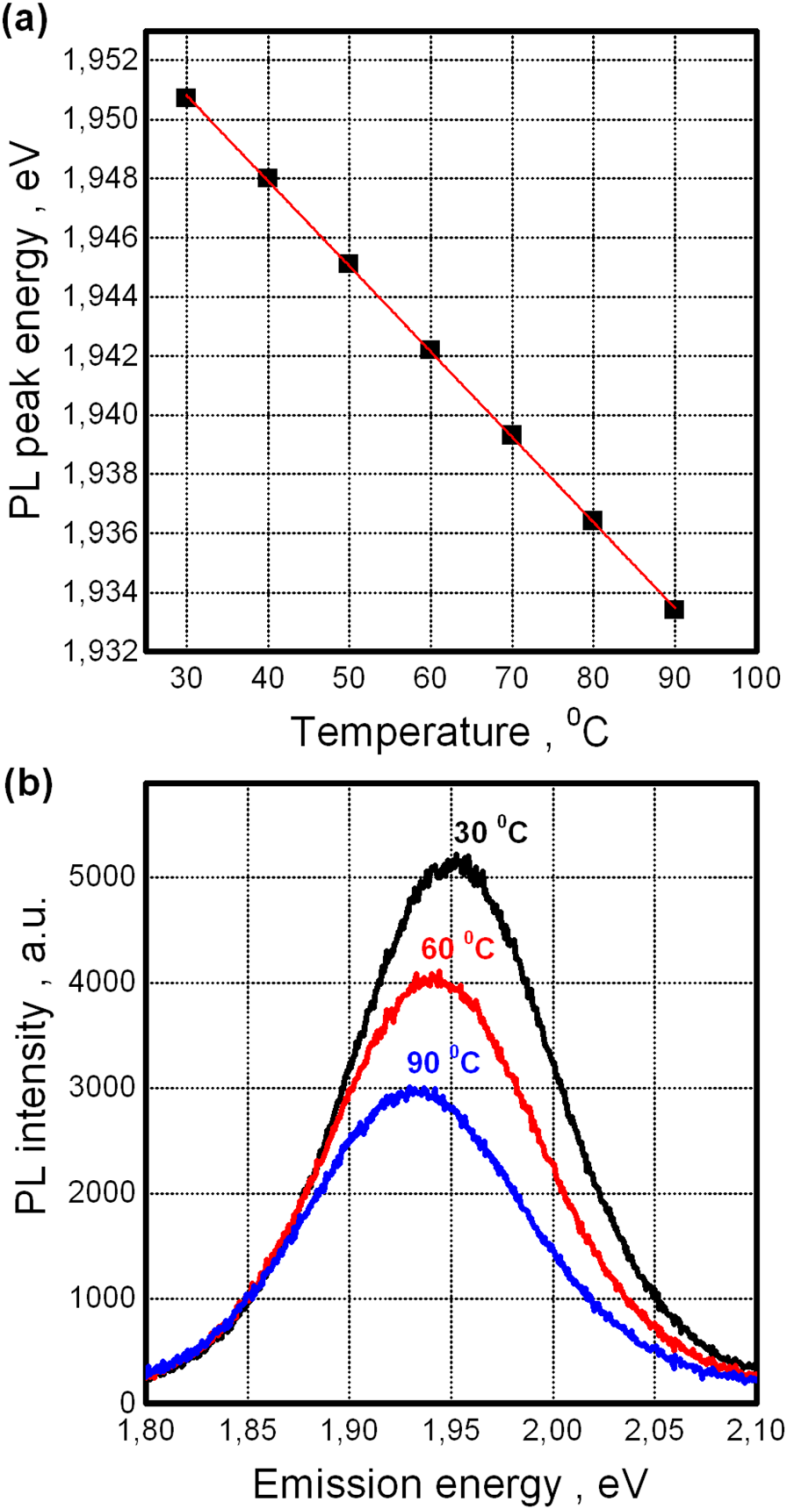
**Photoluminescence response of QDs on dielectric substrate. (a)** Temperature dependence of PL peak position (points) and corresponding Varshni fit (line). **(b)** PL spectra for different temperatures.

**Figure 4:**
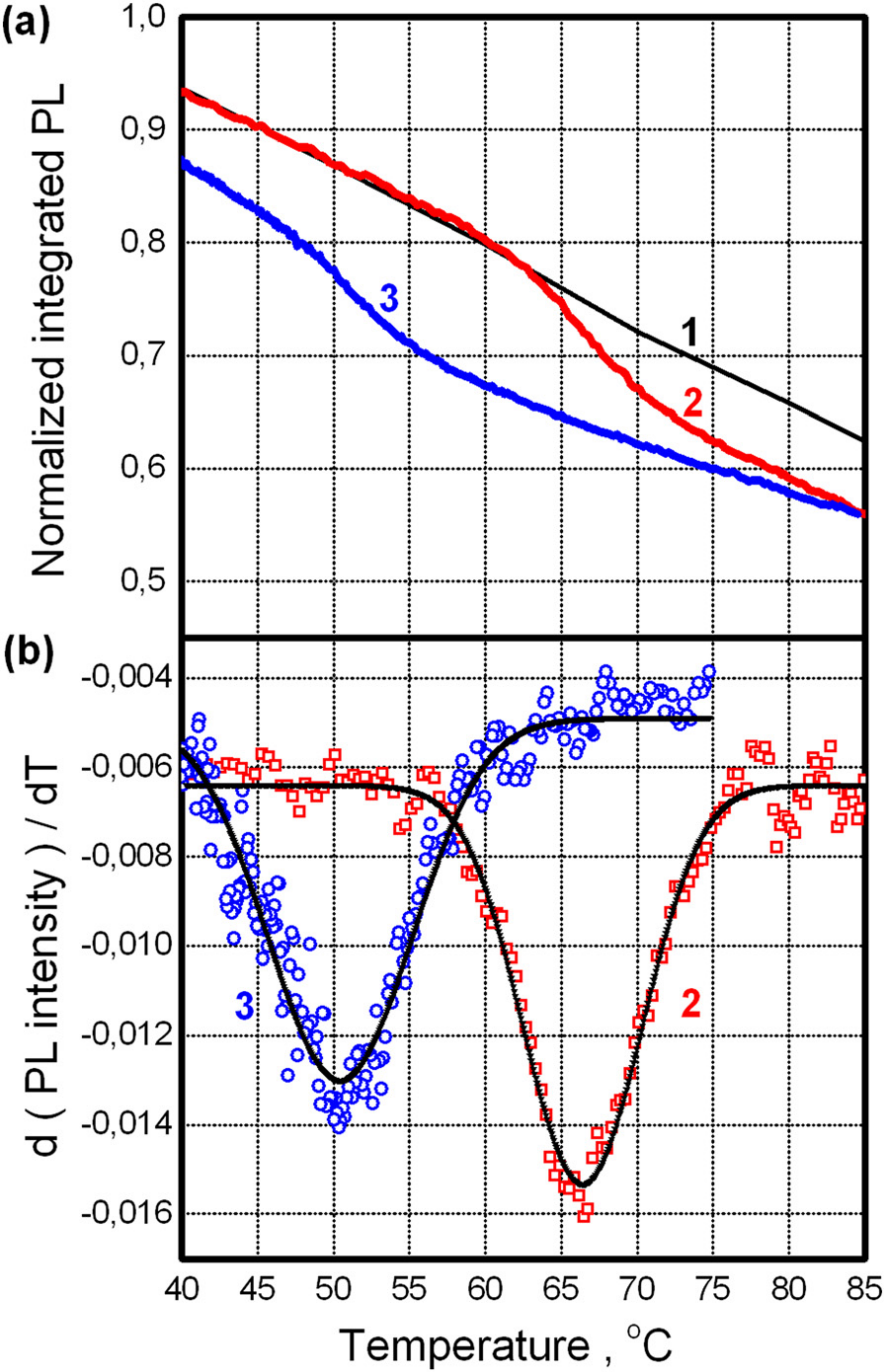
**Photoluminescence response of QDs on VO**_**2 **_**film across metal to insulator transition. (a)** Normalized PL intensity of QDs on dielectric substrate (curve 1) and VO_2_ film under heating and cooling (curves 2 and 3, respectively). **(b)** Corresponding derivatives (symbols) and their Gaussian fit (lines) for upper curves 2 and 3.

Before presenting the main results, we recall the well-known temperature effect on the semiconductor band gap - its narrowing with temperature increase. Concerning CdSe/ZnS QDs, this effect becomes apparent as red shift of PL band [8]. We confirm these observations for QDs deposited on dielectric substrate (namely, the above mentioned sitall). Figure 3a shows temperature dependence of PL peak energy (points) and corresponding fit to empirical Varshni formula [8]. Figure 3b illustrates measured PL spectra of QDs for different temperatures. As can be seen, QDs spectra are characterized by near-Gaussian shape with narrow emission band width of approximately 0.1 eV at half maximum. Note that we will use the term ‘PL intensity’ defined as an integral over whole emission band (so-called integrated PL). Moreover, PL intensity linear decrease is observed with temperature when QDs are placed at the dielectric substrate. This case is shown for normalized PL intensity in Figure 4a, curve 1.

On the contrary, when QDs are placed on the VO_2_ film, PL intensity dependence on temperature deviates from the above mentioned behavior in some temperature ranges: for heating as well as cooling branches. It is shown in Figure 4a, curves 2 and 3, respectively. Across these temperature ranges, PL signal from QDs undergoes noticeable drop and rise for heating and cooling, respectively. Using PL vs. T dependence for the dielectric substrate as a baseline (curve 1), one can estimate a relative PL change as approximately 10% in the above mentioned temperature ranges. To quantify the temperature parameters for these ranges, first derivatives of PL signal were found and they were fitted by Gaussians - see Figure 4b. The extracted parameters are listed in Table 1 including those from electrical measurement. It should be stressed that these data belong to the same VO_2_ film.

**Table 1:** A comparison of characteristic temperatures obtained by different methods

**Method**	**MIT under heating, °C**	**Transition width (FWHM), °C**	**MIT under cooling, °C**	**Transition width (FWHM), °C**	**Hysteresis loop width, °C**
Resistance	68.8	5.4	58.2	4.4	10.6
Photoluminescence	66.4	7.8	50.4	9.5	16
Reflectance at 385 nm	69.7	8.6	50.9	6.0	18.8

A qualitative observation should be added for further discussion. If a VO_2_ film with strong MIT was annealed at 300°C in the air before QD deposition, then the above features in PL response did not appear. At the same time, the annealing dramatically restricted MIT resistance change in that film due to VO_2_ conversion into higher oxides. Therefore, we can refer observed PL features (with their proper parameters) to MIT origin in VO_2_.

**Figure 5:**
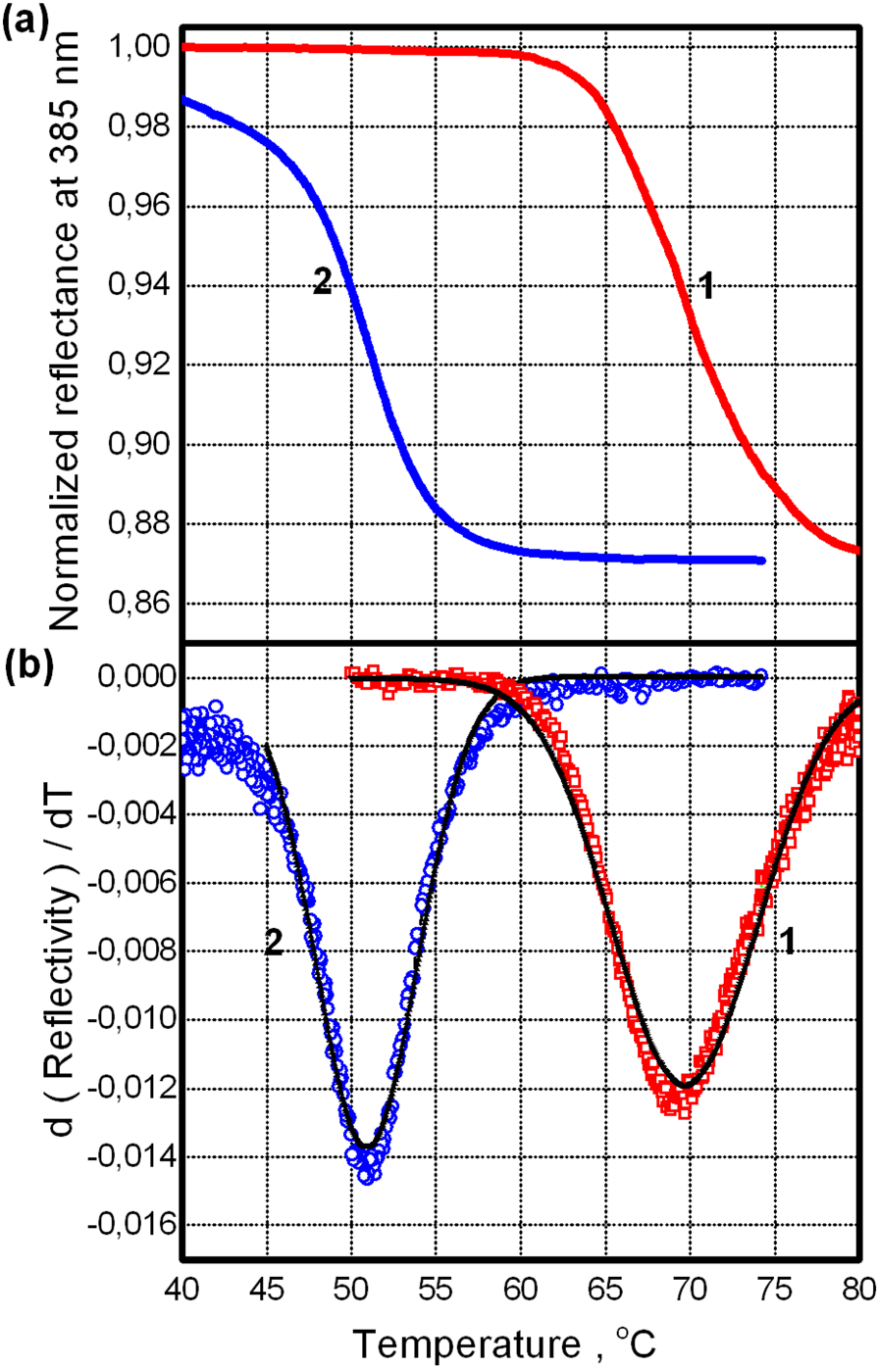
**Relative reflectance response of VO**_**2 **_**film across metal to insulator transition. (a)** Normalized reflectance at wavelength of 385 nm for heating and cooling (curves 1 and 2, respectively). **(b)** Corresponding derivatives (symbols) and their Gaussian fit (lines).

To clarify a mechanism of such behavior, first of all, we have examined purely the optical mechanism. In the PL measurements on our QDs, the two specific wavelength ranges are involved which are centered at 385 nm (excitation) and 630 nm (emission peak). Relative diffuse reflectance at both wavelengths is measured from the VO_2_ film without QDs when a temperature is crossing the MIT region. A reflectance was recorded by spectrophotometer equipped with an integrating sphere. It is revealed that there was no change in the reflectance at 630 nm (within approximately 1% accuracy). However, a noticeable change of approximately 10% in relative reflectance at 385 nm was observed under phase transition - see Figure 5a. It correlates with refractive index modulation in near-UV wavelength range across MIT [9]. Using a differentiation and Gaussian fitting for reflectance vs. temperature curves, characteristic parameters have been found. The results of this procedure are illustrated in Figure 5b for heating and cooling branches. In light of this finding, one may propose the following optical explanation for observed features in PL response of QDs atop the VO_2_ film. First, for our approximately 50-nm thick QD layer, nearly 50% of excitation intensity at 385 nm is absorbed under its forward propagation through this layer [10]. Second, if this QD layer is placed atop reflective surface (in our case VO_2_), a backscattered excitation light may be additionally absorbed by the QD layer. Obviously, it will lead to PL increase from QDs. Finally, when a temperature rises over MIT range, then the reflectance decreases (Figure 5a) and the PL intensity will decrease simultaneously (and vice versa under cooling).

**Figure 6:**
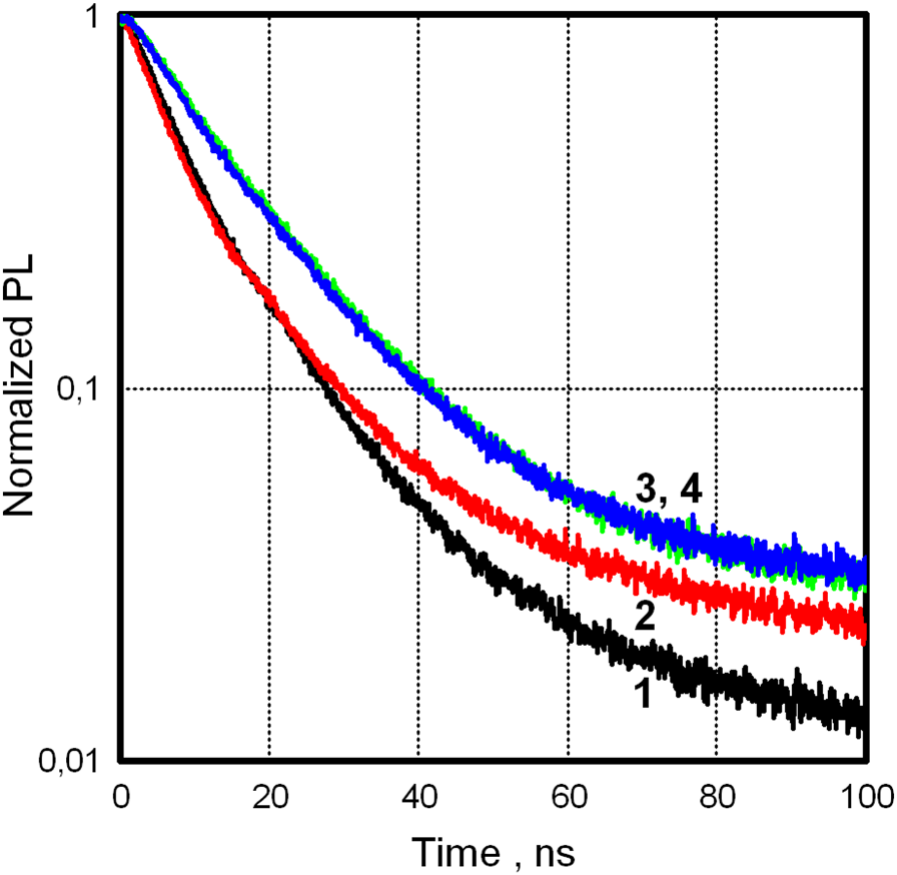
**Normalized PL decay curves for QDs deposited on different substrates.** Vanadium dioxide film, curves 1 and 2; dielectric (sitall), curves 3 and 4. PL transients were measured at room temperature (curves 1 and 3) and at ≥75°C (curves 2 and 4).

This optical consideration meets a support in our measurements of time-resolved PL of QDs on the VO_2_ film. Time-resolved PL spectroscopy is a sensitive method to study an influence of surroundings on radiative processes in QDs. In our case, it is of interest to reveal whether strong change of free electron concentration in VO_2_ across MIT affects PL dynamics in deposited QDs. Figure 6 displays normalized PL decay curves in semi-logarithmic scale obtained with the use of apparatus impulse response function. Two sets of decay curves are shown for QDs deposited on the dielectric and VO_2_ film. Both sets are taken at RT and increased temperature above MIT. To quantify PL lifetimes in the case of nonexponential relaxation, biexponential deconvolution analysis was used. Then, average lifetimes were calculated taking into account the deconvolution parameters for each exponent. For the dielectric substrate, we found mean lifetimes of 14.95 and 14.43 ns at RT and 75°C, correspondently. For the VO_2_ film, they were 10.56 and 10.86 ns at RT and 77°C, correspondently. We see that no noticeable change of PL lifetime of QDs on VO_2_ is recorded in the course of metal to insulator transition when a carrier concentration is changed more than 2 orders of magnitude according our resistance data. Taking into account the direct relation between PL lifetime and steady-state PL intensity in QDs, we can exclude the role of intrinsic interaction between QDs and VO_2_ in the abovementioned PL features during MIT. Two such intrinsic mechanisms are known for QDs in literature: energy and charge transfer from/to excited QDs. The above time-resolved PL data may be an evidence against significant contribution of these mechanisms to PL response of our hybrid structure. This could be expected taking into account the i) out-of-resonance condition between QD's emission energy of approximately 2 eV and plasmon energy lower than 1 eV in metallic phase of VO_2_ (negligible energy overlap) and the ii) QD's thick shell (approximately 2 nm) that is blocking charge carrier exchange.

As can be seen in Figure 6, QDs on the VO_2_ film exhibit higher PL decay rate than that on the dielectric substrate irrespective of temperature. Though this feature is out of scope of the present study, let us touch upon the question. It is noted in the ‘Methods’ section that QD layers were deposited atop substrates by spin-coating from toluene solution followed by baking at approximately 100°C for 1 h in the air. The latter treatment is aimed to decrease a degradation of QD's PL properties in the course of subsequent heating/cooling measurement cycles. This annealing of QDs deposited on VO_2_ led to more pronounced PL decrease (more than twofold) against that for simple drying at RT. It should be stressed that there is no significant PL decrease when the same preparation was done on the dielectric substrate. We can assume that PL efficiency of QDs on VO_2_ is quenched due to well-known catalytic activity of vanadium oxides.

Let us discuss characteristic temperatures obtained by three methods. As can be seen in Table 1, satisfactory coincidence between characteristic temperatures exists for insulating to metallic phase change under heating (within our temperature uncertainty of ±1°C). For reverse phase transition (cooling), there is a prominent discrepancy between the temperature derived from electrical measurement and those for both optical characterization methods.

Only few works are devoted to combined experimental study of MIT phenomenon including electrical and optical characterization methods. The work [11] presents simultaneous optical and electrical measurements of MIT in a two-dimensional nanostructured VO_2_ film and shows that there is an offset between the effective optical and electrical switching temperatures. The authors proposed that this offset arises because the hysteresis in optical transmittance is determined by the relative fraction of grains in semiconducting state, while the hysteresis in electrical resistance is governed by the evolution of a continuous percolation path. It is obvious that this explanation is not valid for thick polycrystalline films.

The authors of [12] observed the difference in MIT temperatures probing by electrical (resistance) and optical (reflectance in IR) measurements. However, the cited authors analyzed the results only for heating branch. It was found that switching temperature derived from resistance data has significant shift against optically derived temperature. To clarify the origin of observed difference, the authors revised their electrical measurements in terms of conductance instead of resistance and found much less discrepancy. They concluded that conductance instead of resistance is a more suitable characteristic in comparison with optical data. Note that the same mathematical treatment (see above) for extraction of characteristic temperature from electrical measurement was used in the cited work. Then, it is simple to show from relation *σ* = *R*^-1^ that *d*(Log *σ*)/*dT* = -*d*(Log *R*)/*dT*, where *σ* is the conductance and *R* is the resistance. Therefore, such substitution cannot change the final result. Our detailed consideration of the cited work is aimed to verify the validity of resistance-based approach used in our study.

Thus, we observe the nonequivalence of MIT trajectories under heating and cooling that is manifested by the difference between characteristic temperatures derived from electrical and optical data. We assume that more systematic and sophisticated experimental studies are needed to clarify an underlying mechanism.

## Conclusions

We have probed the phase transition in the vanadium dioxide film decorated with colloidal quantum dots measuring a temperature dependence of PL response. Characteristic temperatures derived from PL measurement are compared with those obtained by standard conductivity method. The reflectance dependence on temperature taken at PL excitation wavelength points out that PL mimics a reflectance change in VO_2_ across MIT. No evidence was observed in favor of strong interaction between QDs and VO_2_ caused by MIT.

## Competing interests

The authors declare that they have no competing interests.

## Authors’ contributions

SNK proposed the conception and drafted the manuscript. ABC performed experiments, analyzed the data, and helped in drafting the present manuscript. GBS revised the manuscript critically. All authors read and approved the final manuscript.
